# Monitoring the Effects of Hemicellulase on the Different Proofing Stages of Wheat Aleurone-Rich Bread Dough and Bread Quality

**DOI:** 10.3390/foods10102427

**Published:** 2021-10-13

**Authors:** Boyu Tian, Chenxia Zhou, Dongxiao Li, Jiawei Pei, Ailiang Guo, Shuang Liu, Huijing Li

**Affiliations:** 1 College of Food Science and Technology, Hebei Agricultural University, Baoding 071001, China; tianboyu1122@163.com (B.T.); 18731229633@163.com (C.Z.); peijw14@163.com (J.P.); ailiangguo2021@163.com (A.G.); shuangliu202109032@163.com (S.L.); 2 Key Laboratory of Crop Growth Regulation of Hebei Province, College of Agronomy, Hebei Agricultural University, Baoding 071001, China; lidongxiao.xiao@163.com

**Keywords:** hemicellulase, wheat aleurone-rich bread quality, intermolecular forces, wheat gluten, proofing stage

## Abstract

This study investigated the effects of a hemicellulase dosage (20, 40, and 60 mg kg^−1^ of flour) on the bread quality and rheological properties of wheat aleurone-rich flour. The results showed that hemicellulase could soften dough and improve extensibility. At the optimum hemicellulase dosage (40 mg kg^−1^ of flour), the bread specific volume increased by 40.91% and firmness of breadcrumb decreased by 104.57% compared to those of the control. Intermolecular forces indicated that the gluten network during the proofing was mainly strengthened via disulfide bonds, hydrophobic interactions, and hydrogen bonds but not through ionic bonds after hemicellulase addition. Fourier infrared spectroscopy indicated that the hydrolytic activity of hemicellulase catalyzed the transition from α-helix to β-sheet, which verified that viscoelasticity of gluten was enhanced at a dosage of 40 mg kg^−1^ of flour. These results suggested that hydrolyzation of hemicellulase contributed to the structural of gluten changes, thereby improving the quality of wheat aleurone-rich bread.

## 1. Introduction

It is gradually evident that whole grains play an important role in the prevention of chronic diseases [1,2]. Numerous epidemiological studies have demonstrated that whole grains consumption increase may be related to a low incidence of type II diabetes, cardiovascular diseases, and cancer [3,4,5]. Although the mechanisms underlying of these protective effects have not been completely clarified, the concept that major phytochemicals against the nutritional and metabolic diseases were located in the bran layers has been increasingly recognized and accepted [6]. Multitudinous researches have also described that the beneficial function of whole grains intake may depend on the synergic actions of multiple compounds in bran, particularly in the aleurone layer [7,8].

Wheat aleurone layer is a single-layer histological structure composed of living cells which consisted of the intracellular medium or cytoplasm and thick non-lignified cell wall [9]. The cytoplasm of aleurone cell is characterized by large amounts of mineral (around 40–60% of the total wheat grain mineral content), protein (approximately 15% of the total wheat kernel protein content), B vitamins, plant sterols, and phytates [8,10]. Meanwhile, the wheat aleurone cell wall includes 65% relatively linear arabinoxylan with a low ratio of arabinose-to-xylose, 29% β-glucan, and a few proteins [11,12]. Due to its nutritional enrichment and potential health effects, as well as the development of novel milling techniques, wheat aleurone layer or wheat aleurone-rich fraction has been gradually used as a novel raw material to replace bran in the field of food processing. Considerable application work has been carried out in researching and developing a whole grain bread with white bread characteristics [13]. It shows that bread with a 20% replacement of wheat aleurone flour has the nutrition comparable to whole wheat bread, but flavor and appearance were closer to bread made with refined wheat flour. Blanka et al. [14] also observed that wheat aleurone-rich flour as a nutritionally valuable milling product caused lower reduction in the baking quality of bread wheat blends than the whole wheat flour. However, the wheat aleurone layer bread still displayed a smaller specific volume and coarser texture than commercial white bread, which may continue to limit its appeal to consumers and have a negative impact on the wheat aleurone foods market. To the best of our knowledge, the most common approach to alleviate or eliminate the negative effects of non-starch polysaccharides (NSPs) on bread quality involves the application of enzymes during bread making.

Hemicellulase usually refers to a group of enzymes which could synergistically hydrolyze hemicellulose; the hemicellulytic system mostly involves endo- and exo-1,4-β-xylanase, β-arabinofuranosidases, α- and β-galactosidases, β-xylosidases, endo-1,4-β-mannanase, and β-mannosidases [15,16]. Hemicellulase has been commonly used in the breadmaking to improve bread loaf volume and crumb structure. It is known that arabinoxylans (AX) exert an important role in the formation of gluten networks, and it has been reported that the water-unextractable arabinoxylans (WUAX) had a negative effect on bakery products, whereas high molecular weight water-extractable arabinoxylans (WEAX) showed a beneficial trend [17,18]. Hemicellulase acts by cleaving AX chains, thus causing redistribution of water from WUAX to gluten and makes the water available for gluten hydration [19]. Moreover, high-molecular-weight WEAX produced by hydrolysis could further promote the formation of gluten network, thereby strengthening the gas-holding capacity of dough [20]. Matsushita et al. [21] examined the effect of baking enzymes (α-amylase and hemicellulase) on the quality of bread making, and confirmed that the increase in dough volume was directly associated with the decrease in natural detergent fiber and hemicellulose content caused by the hemicellulase hydrolyzed the dietary fibers. Altınel et al. [22] found that whole wheat bread made by the addition of 0.01% (*w/w*) hemicellulase had significantly higher loaf volume and overall acceptability than those of the control group, meanwhile, they also indicated that addition of an optimized hemicellulase dosage to dough led to deteriorating in rheological properties. Those results might mean that the decrease in mechanical strength of fiber-rich dough caused by hemicellulase addition had a positive correlation with the increase in bread volume. In contrast, the deterioration of rheological properties of dough had a conflict with the previous inference that hydrolysis of NSPs improved gluten network. However, there was still a lack of complete information to explain the contradiction resulted in the addition of hemicellulase between the deterioration in rheological properties of dough and the improvement in bread quality.

In light of the above, this study sought to assess the performance of hemicellulase addition on the bread quality (textural properties and specific volume) and rheological properties of wheat aleurone-rich dough. More specifically, in order to elucidate the differences in the effects of hemicellulase on dough rheological properties and bread quality, the gluten quality (content of wet gluten and gluten index) and intermolecular forces (free sulfhydryl, disulfide bond, ionic bonds, hydrogen bonds, and hydrophobic interactions) at each stage of bread dough proofing were investigated. Furthermore, subunits molecular weight and secondary structure of gluten at typical proofing stages and different hemicellulase dosages were also determined in combination with the changes in the above indicators.

## 2. Materials and Methods

### 2.1. Materials

The wheat (Gaoyou 2018) used in the present study was purchased from Xinhong Grain Trade Co. Ltd. (Baixiang, China). The clean wheat was subjected to 13% moisture content, 1.5% secondary humidification amount, and debranned by horizontal rice mill (Huachang Grain and Oil Machinery Co. Ltd., Taizhou, China) for 80 s. According to our previous study, the aleurone layer accounted for the highest proportion of wheat grains with those processing conditions. The components of the debranned wheat were determined according to method modified by Chen et al. [12] and Hemery et al. [23]. The proportions of outer pericarp, intermediate layer, aleurone layer, germ, and endosperm of the debranned wheat kernel were 0.66 ± 0.04%, 3.30 ± 0.02%, 9.20 ± 0.06%, 0.03 ± 0.01%, and 86.81 ± 0.05%, respectively. The debranned wheat was milled by a ALMB Buhler laboratory mill (Buhler Grain Inspection Instrument Wuxi Co., Ltd., Wuxi, China), and the wheat aleurone-rich fraction (aleurone layer content was 54.11%, phytate content was 43.29 mg g^−1^) was separated from the wheat flour through a ALMC sifter (Buhler Grain Inspection Instrument Wuxi Co., Ltd., Wuxi, China). The separated wheat aleurone-rich fraction was heated in a DXY-100 KW microwave equipment (Dongxuya Machinery Equipment Co., Ltd., Jinan, China) with microwave output power 700 W for 60 s to inactivate the endogenous enzymes. Subsequently, the stabilized wheat aleurone-rich fraction was ground to yield a particle size of 180 μm and added back to the wheat flour at original proportion (wheat aleurone-rich fraction: wheat flour, 18:82 (*w/w*, 14% moisture basis)). According to T/HBFIA 0011-2020 [24], the blended flour conformed to the standard of wheat aleurone-rich flour (moisture content 12.80%, ash 0.81%, dietary fiber 10.6%, alkylresorcinol content 261.20 μg g^−1^, fatty acid value 66 mg KOH/100 g, sediment content 0.01%, and magnetic metal content 0.001%). The hemicellulase (the hemicellulase activity in the manual was 1300 U g^−1^, and the xylanase activity contained in the hemicellulase was determined to be 35,720 U g^−1^ according to the method reported by Yegin et al. [25]) was purchased from DSM Food Specialties (Delft, The Netherlands). Sugar, salt, butter, and yeast were obtained from a local supermarket. All chemicals used were at a minimum analytical grade.

### 2.2. Bread Baking and Dough Preparation

Breadmaking recipes and procedures were performed with a modified GB/T 35869-2018 [26]. All ingredients, including wheat aleurone-rich flour (200 g), distilled water (130 mL), sugar (12 g), salt (3 g), yeast (4 g), and butter (6 g). Enzyme at dosages of 0, 20, 40, and 60 mg kg^−1^ wheat aleurone-rich flour were tested. The hemicellulase was mixed with wheat aleurone-rich flour by gradient dilution before being used in order to promote its homogenous distribution within the wheat aleurone-rich dough. The ingredients except butter were mixed in a DKM-201 kitchen kneading machine (Diyi Electrical Technology Co., Ltd., Shunde, China) at 70 rpm for 4 min, and then butter was added to the dough and mixed at 150 rpm for 10 min to develop gluten. The dough was placed into an MXF-A fermentation cabinet (Meiying Food Equipment Co., Ltd., Jinan, China) at 30 °C with a relative humidity of 80% for 60 min. During fermentation, sheeting and punching was carried out every 20 min. After first proofing period, dough was divided into small pieces (170 g/each), hand rounded, and then proofed at 30 °C with a relative humidity of 80% for 80 min (second proofing period). The dough was baked at 180 °C for 20 min in a KA401 far-infrared food oven (Chubao Baking Machinery Equipment Co., Ltd., Guangzhou, China).

The 20, 40, and 60 min of proofing time in the first proofing period were marked as stage I, stage II, and stage III, respectively. The 20, 40, 60, and 80 min of proofing time in the second proofing period were recorded as stage IV, stage V, stage VI, and stage VII, respectively. Dough at each stage of the proofing process was collected, some part of fresh dough was used to analyze its properties and the rest was kept lyophilized, then it was ground and stored at 4 °C for subsequent experiments.

### 2.3. Quality Evaluation of the Baking Bread

The bread loaf volume was measured by the rapeseed displacement according to American Association of Cereal Chemists (AACC) Method no. 10-05.01 [27], and the specific volumes of bread were determined from the volume/mass ration and expressed in mL g^−1^. A TMS-Console Texture Analyzer (Food Technology Co. Ltd., Des Moines, IA, USA) equipped with a 20-mm-diameter aluminum probe which was used to determine the firmness of breadcrumb. Bread slices (20 mm in thickness) were removed from the center of bread, and compressed twice to 50% of original height at a speed of 60 mm min^−1^ [28].

### 2.4. Rheological Properties of Dough

Farinograph properties were determined according to AACC Method no. 54-21.02 [29] using a Farinograph-AT equipped with a 300-g stainless steel bowl (Brabender GmbH and Co KG, Duisburg, Germany). The dough properties of water absorption (WA, %), development time (DDT, min), stability time (ST, min), and degree of softening (DS, UF) were recorded for analysis. Extensograph properties were carried out according to AACC Method no. 54-10.01 [30] using an Extensograph-T (Brabender GmbH and Co KG, Duisburg, Germany). The extension (mm), extension area (cm^2^), tensile resistance (BU), and maximum tensile resistance (BU) of dough were summarized at 45, 90, and 135 min.

### 2.5. Gluten Quality Measurements

The content of wet gluten and gluten index was determined by a JJJM54 glutomatic system (Huier Instrument Equipment Co., Ltd., Hangzhou, China), according to AACC Method no. 38-12A [31]. The gluten index is a parameter providing information on both gluten quality and gluten quantity, and it expresses the weight percentage of the wet gluten remaining on a sieve after automatic washing with salt solution and centrifugation.

Gluten index (%) = wet gluten remaining on siese (g)/total wet gluten (g) × 100 (1)

### 2.6. Quantification of the Free Sulfhydryl and Disulfide Bond in Dough

The content of the free sulfhydryl and disulfide bond were determined using modified Ellman’s reagent by colorimetric method [32]. The lyophilized dough powder (400 mg) and 10 mL buffer (3 mM EDTA, 8 M urea, 0.2 M Tris-HCl, pH 8.0) were stirred for 10 min with a magnetic stirrer at 400 rpm, then centrifuged at 3500× *g* for 10 min and the supernatant was collected. For free sulfhydryl, 0.1 mL of Ellman’s reagent (10 mM 5,5-dithio-bis (2-nitrobenzoic acid), 3 mM of EDTA, and 0.2 M of Tris-HCl, pH 8.0) was added to 4 mL of supernatant, and the mixture was incubated for 20 min at 25 °C in the dark. For total sulfhydryl, 0.4 mL of supernatant, 1.6 mL of buffer, and 0.04 of mL β-mercaptoethanol were mixed and then incubated for 1 h at 25 °C. After an additional 1 h incubation with 4 mL 12% (*w/v*) trichloroacetic acid (TCA), the reaction tubes were centrifuged at 3500× *g* for 10 min. The precipitate was twice resuspended in 5 mL 12% TCA and centrifuged to remove β-mercaptoethanol. The precipitate was dissolved in 4 mL of buffer, 0.1 mL Ellman’s reagent was added, and the mixture was incubated for 20 min at 25 °C in the dark. The absorbance at 412 nm of the reaction solution was all measured against a Ellman’s reagent blank. The content of free and total sulfhydryl groups (μmol g^−1^) in the dough were calculated by the standard curve of L-cysteine. Y = 0.0864X − 0.0008 (r^2^ =0.999), X was the absorbance at 412 nm, Y was the content free sulfhydryl (μmol g^−1^).

The content of disulfide bond = (content of total sulfhydryl − content of free sulfhydryl)/2(2)

### 2.7. Determination of Non-Covalent Bonds in Dough

Chemical interactions were determined using the method described by Yao et al. [33] with some modification. Briefly, buffers (prepared in 0.05 M phosphate buffer, pH 7.0) were selected and used to disrupt certain kinds of bonds in the protein structure as follows: 0.05 M of NaCl (SA), 0.6 M of NaCl (SB), 0.6 M of NaCl + 1.5 M of urea (SC), and 0.6 M of NaCl + 8 M of urea (SD). Each freeze-dried dough (400 mg) was dispersed in 10 mL of each buffer in a STSRH-200 homogenizer (Suo Yan Mechanical and Electrical Equipment Co., Ltd., Shanghai, China) for 2 min. The resulting suspension was stirred at 25 °C for 1 h and centrifuged at 10,000× *g* for 20 min. The concentration of protein in the supernatant was determined by the Bradford method. The result was determined from the soluble protein quality/volume of supernatant ration and expressed in mg mL^−1^. The contribution of ionic bonds was expressed by the difference in protein content dissolved in SB and SA; the contribution of hydrogen bonds was expressed by the difference in protein content dissolved in SC and SB; and the contribution of hydrophobic interactions was expressed by the difference in protein content dissolved in SD and SC.

### 2.8. Sodium Dodecyl Sulphate-Polyacrylamide Gel Electrophoresis (SDS-PAGE) Analysis

SDS-PAGE was performed using 10% separating gel (pH 8.8) and 8% stacking gel (pH 6.8). Each sample (2 mg) was stirred in 1.0 mL of extraction buffer (0.1 M Tris-HCl, pH 6.8, including 4% (*w/v*) SDS, 10% (*v/v*) β-mercaptoethanol, 20% (*v/v*) glycerol, and 0.005% (*w/v*) bromophenol blue) for 24 h. For non-reduced proteins, extraction buffer did not contain β-mercaptoethanol. Samples were heated for 5 min at 100 °C, and then centrifuged for 20 min at 10,000× *g*. The protein amount of the loaded samples was 10 μg per channel, which was quantitated by the Bradford method. The running buffer (pH 8.3) consisted of 25 mM of Tris, 1% SDS, and 192 mM of glycine. The electrophoresis was performed at a voltage of 80 V in the stacking gel but 160 V in the separating gel. When electrophoresis was completed, gels were fixed in a fixing solution (ethanol: acetic acid: deionized water, 5:1:4 (*v/v/v*)) for 2 h. Afterwards, gels were stained (0.25% (*w/v*) Coomassie blue in the solution containing ethanol, acetic acid, and deionized water (5:1:4, *v/v/v*)) for 3 h and then destained in the solution containing ethanol, acetic acid, and deionized water (20:7:63, *v/v/v*) [34].

### 2.9. Determination of Molecular Weight and Distribution of Gliadins

The molecular weight and distribution of gliadins were determined according to the method of Wang et al. [35] with some modifications. A freeze-dried sample (5 mg) was suspended in 2 mL of sodium phosphate buffer (0.05 M, pH 6.9) and stirred for 2 h with a magnetic stirrer at 400 rpm. The supernatant was gathered after centrifugation for 20 min at 10,000× *g* then filtered through 0.22-μm filters before Gel permeation chromatography (GPC) analysis. P230 GPC system (Elite Instruments Co., Ltd., Elk Grove Village State, IL, America) was using a BIOSEP SEC-4000 (300 × 7.8 mm) column (Phenomenex Co., Ltd, Torrance, CA, America). Each sample (20 μL) was injected into the column and ran for 30 min with a flow rate of 0.5 mL min^−1^ at 40 °C. The elution solvent was 45% acetonitrile in water (*v/v*) containing 0.1% trifluoroacetic acid (*v/v*). The eluted proteins were detected by UV absorbance at 220 nm. Protein molecular weight was calculated by the standard curve of peptides. Y = −0.2302X + 6.8479 (r^2^ = 0.999), Y was the logarithm of the molecular weight of the peptide, X was retention time.

### 2.10. Determination of the Secondary Structures of Gluten

The secondary structure of lyophilized gluten powder samples was evaluated by a Fourier Transform Infrared (FTIR) spectrometer (Nicolet IS10, Nicolli Instruments, Madison, WI, USA). Potassium bromide powder (150 mg) dried to a constant weight was ground in an agate mortar. The ground powder was placed into the groove of a tablet press and manually pressurized to prepare the samples. The obtained potassium bromide reference had to be free of cracks and transparent, and it was placed in the slide slot, and scanned in the infrared spectrometer as a blank. Each sample was determined by the following steps, with one replicate. Only one potassium bromide tablet was prepared for each sample [33]. Samples of gluten (2.0 mg) and potassium bromide powder (150 mg) were mixed and ground in an agate mortar, and the subsequent operation was the same as that of the blank production. Infrared spectra (128 scans) were recorded with a resolution of 4 cm^−1^ at room temperature in the region 400–4000 cm^−1^. Relative data points were exported by Omnic software (Omnic Specta 7.1, Thermo Fisher Scientific Inc., Waltham, MA, USA) and Peak Fit software (PeakFit v4.12, SeaSolve Software Inc., San Jose, CA, USA) was used to analyze the infrared (IR) spectrum. Calculating the second derivative and fitting the Gaussian curve (until the correlation coefficient r^2^ remains unchanged) to predict the content of secondary structure. The peak identification of the amide I band (1600–1700 cm^−1^) was relatively mature, with 1652–1660 cm^−1^ corresponding to α-helices, 1600–1644 cm^−1^ and 1685–1700 cm^−1^ corresponding to β-sheets, 1660–1685 cm^−1^ corresponding to β-turns, and 1644–1652 cm^−1^ corresponding to random coils [36]. The secondary structure components of samples were calculated using the ratio of the corresponding area to the total amide I band area.

### 2.11. Statistical Analysis

The data were statistically performed using SPSS 21.0 (IBM, Armonk, NY, USA), and the results were presented as the mean ± standard deviation (SD). One-way analysis of variance (ANOVA) and Duncan’s test were applied, and significant differences in mean values were compared based on the 95% confidence interval (*p* < 0.05). The data were depicted using GraphPad Prism 8.0.1 (GraphPad Software, San Diego, CA, USA).

## 3. Results and Discussion

### 3.1. Effect of Hemicellulase Addition on Bread Quality

**Figure 1:**
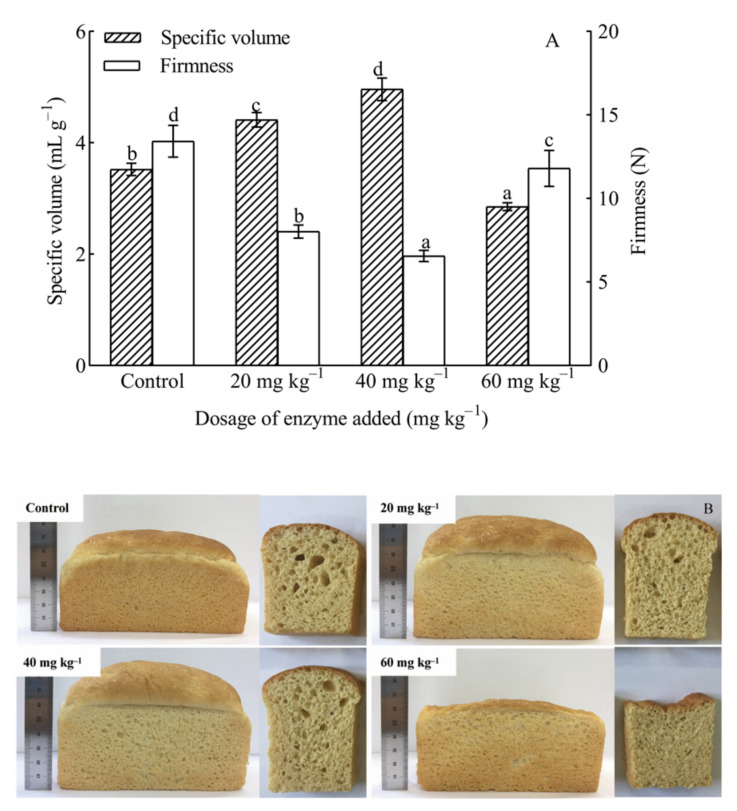
The effect of adding hemicellulase on the specific volume and crumb firmness of bread (**A**). Photographs and scanned images of various breads and their crumbs (**B**). Different letters above the bars with same pattern indicate significant difference between the different dosage of hemicellulase (*p* < 0.05).

One of the most important parameters in the evaluation of breadmaking quality is the specific volume. Figure 1A illustrated that the specific volume of bread gradually increased with increasing the hemicellulase dosage from 20 to 40 mg kg^−1^ of flour. Compared with the control bread, the specific volume with hemicellulase at a dosage of 40 mg kg^−1^ of flour added had increased by 40.91%. Afterwards, the decrease in specific volume was obtained. When hemicellulase was used at a dosage of 60 mg kg^−1^ of flour, the proof volume of the dough was the largest, but it collapsed during baking. Similarly, Oliveira et al. [37] and Yegin et al. [25] also found that the addition of xylanases at optimum level into bread recipes provided an increase in specific volume of breads, but high dosage of xylanase caused a setback in bread specific volume. They indicated that the impact of xylanases on the loaf volume was not only governed by the production of enzyme-solubilized AX, but also by the removal of WUAX. Increased AX solubilization had been associated with increased loaf volume, because soluble AX was likely to increase the viscosity of the liquid film around the gas cells and enhance the gas retention capacity of dough. However, the further pyrolysis of AX affected the consistency of the dough and more stick doughs were obtained, resulting in reduced air cell stability and a smaller bread volume.

Firmness of breadcrumb is very important for the consumer acceptability and perception. As shown in Figure 1A, firmness of breadcrumb was inversely proportional to the specific volume of bread with the increase in the enzyme dosage. The firmness of breadcrumb was the lowest when the specific volume of bread was the highest at a dosage of 40 mg kg^−1^ of flour. Similar results of achieving a high specific volume despite a low initial firmness had been obtained in another study [38]. It was also worth noting that the specific volume of bread addition with 60 mg kg^−1^ of flour was lower than that of the control, but it provided lower firmness of breadcrumb than that of the control. This was thought to be caused by the low peak viscosity and setback viscosity (Table S1) which would evidently decrease the firmness of breadcrumb [28,39]. Overall, 40 mg kg^−1^ of flour could be determined with the optimum enzyme addition amount.

### 3.2. Effect of Hemicellulase Addition on Farinograph Properties of Dough

The effects of hemicellulase on farinograph properties of wheat aleurone-rich dough are presented in Table 1. It was clearly observed that there was no significant change in WA of the wheat aleurone-rich dough by the addition of hemicellulase as compared to that of the control (*p* < 0.05). A similar observation was obtained by Burak et al. [20], and the addition of a blend of hemicellulases, consisting mainly of endoxylanase, did not produce any change in WA of whole wheat dough. It had been indicated that hydrolysis of larger NSPs into smaller ones with a low water-absorbing capacity results in reduced WA of dough; however, WEAX and ferulic acid (FA) released from hydrolysis could promote gluten and free water to form gluten network. Therefore, it could be speculated that hemicellulase had no influence on the WA of aleurone-rich dough when the above effects reached equilibrium. In the present study, DDT exhibited a decreasing trend and it reached to almost a constant value when 40 mg kg^−1^ of flour enzyme dosage was used. At an enzyme dosage of 60 mg kg^−1^ of flour, DDT significantly declined (*p* < 0.05). From this phenomenon, it could be inferred that a high dosage of hemicellulase exceeded the threshold for extensive hydrolysis of AX and large amounts of WUAX begin to convert into WEAX [40]. The water-absorbing capacity per gram of WEAX and WUAX in wheat were reported as 6.3 g of water and 9.9 g of water, respectively [41]. Therefore, gluten could be hydrated to form gluten network, thereby shortening DDT of dough. Generally, low values of DS together with a high value of ST indicated better handing ability and higher resistance to mechanical stress. In the present study, the ST and DS were very similar to those of the control dough at hemicellulase dosages of 20 and 40 mg kg^−1^ of flour. However, when the enzyme dosage was 60 mg kg^−1^ of flour, there was a sharp change as compared to that of the control dough. It had been suggested that application of overdosage of endoxylanases resulted in excessive hydrolysis of AX [25]. With the enrichment of free water, dough consistency dropped dramatically and sticky doughs were obtained [25]. This effect showed that, when the addition amount of hemicellulase was 60 mg kg^−1^ of flour, it was very likely to induce excessive hydrolysis of hemicellulose, adversely affecting the stability of wheat aleurone-rich dough.

According to farinograph data, it could be observed that addition of hemicellulase leading to decrease mechanical resistance of dough. High-dosage hemicellulase could accelerate the hydrolysis rate of NSPs and promote the formation of gluten network, but the negative effect of excessive hydrolysis was gradually displaying in the late processing. When the dosage of enzyme was 60 mg kg^−1^ of flour, the notable decrease in bread quality also confirmed the above speculation. However, the difference between the improvement of bread quality and the deterioration in the farinographical behaviors of the dough when the enzyme was added at 20 and 40 mg kg^−1^ of flour still needed to be evaluated by combining these results of extensograph properties of dough.

### 3.3. Effect of Hemicellulase Addition on Extensograph Properties of Dough

**Figure 2:**
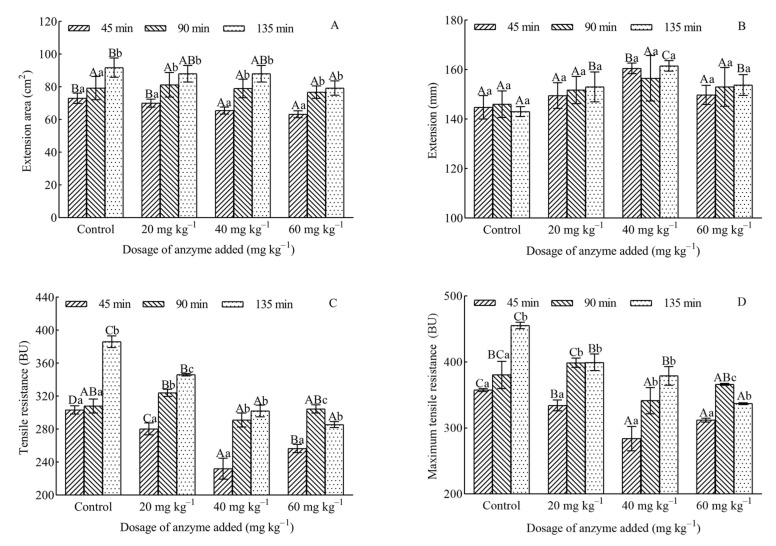
Effect of different dosages of hemicellulase preparations on extensograph properties of dough. Extension area (**A**), Extension (**B**), Tensile resistance (**C**), Maximum tensile resistance (**D**). Different lowercase letters indicated that there were significantly different between the same enzyme dosage and different resting time between the different resting time (*p* < 0.05), and different capital letters indicated that there were significantly different between the same resting time and different enzyme dosages between the different dosage of hemicellulase (*p* < 0.05).

As illustrated in Figure 2A,C,D, the extension area (A), tensile resistance (R), and maximum tensile resistance (Rm) of dough prepared with or without hemicellulase increased with the prolonging in resting time from 45 to 135 min (regardless 90 min). At 135 min resting time, the highest A, R, and Rm were found in the control group (91.75 cm^2^, 386.00 BU, and 455.50 BU, respectively). Wheat aleurone-rich dough supplemented with a dosage of 60 mg kg^−1^ of flour had the lowest A, R, and Rm among all samples tested in the present study. As shown in Figure 2B, the addition of hemicellulase could improve the extension (E) of dough, but there was no statistically substantial increase in E for any resting time of dough. Those were similar to the findings of Altınel et al. [22] who indicated that incorporation of hemicellulase into whole wheat dough formulation led to a reduction in the R value. Nishitsuji et al. [42] pointed out that extensibility of dough decreased and R of dough increased with accumulating in WUAX content of flour. Water unextracted solids, mainly containing WUAX, negatively affected the dough properties because they had a high water-binding capacity, which was reflected in a high Rm and a small extensibility at Rm of dough [43]. From another perspective, Ma et al. [44] reported that WUAX prevented the formation of some disulfide bonds and weakened the viscoelasticity of gluten. Therefore, it was more likely to expect dough with low R and high E by the addition of hemicellulase into bread dough formulation due to the breakdown of AX by these enzymes. Combining the enhancement of the bread quality at enzyme dosages of 20 and 40 mg kg^−1^ of flour, it could be inferred that the reduction in R and A of dough caused by the hydrolytic activity of hemicellulase was beneficial to the improvement handling ability of dough and bread loaf volume. However, it was noteworthy to mention that R and Rm of dough addition with a dosage of 60 mg kg^−1^ of flour at a 135 min resting time were significantly lower than that of dough at a 90 min resting time. At a 135 min resting time, the E of dough with high dosage (60 mg kg^−1^ of flour) was significantly smaller than that of the low dosage (40 mg kg^−1^ of flour) group, even though the A and R of dough had no significant change (*p* < 0.05). Those results suggested that the gluten network became more open and easier to fracture at high level of hemicellulase, which made it different to the further improvement in the bread quality [45].

Overall, extensograph data showed that the resting time of dough and the hemicellulase dosage had a synergistic effect on the quality of dough. Although the resting time of the extensographical test was closer to the bread dough proofing time, the effects of other components of breadmaking ingredients and dough processing on the bread quality were not reflected. Therefore, intensive evaluation of hemicellulase had to be performed by combining rheological results with the changes of various indicators in the actual processing of bread dough.

### 3.4. Effect of Hemicellulase on Content of Wet Gluten and Gluten Index during Dough Proofing

**Figure 3:**
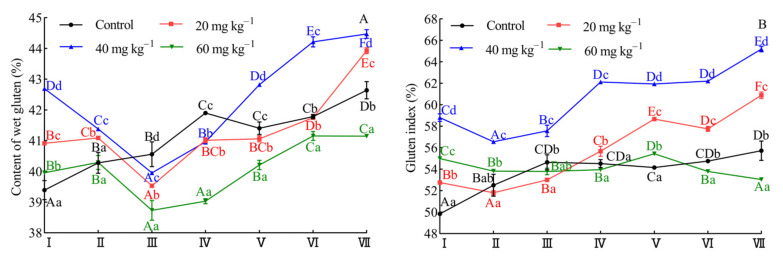
The effect of hemicellulase on content of wet gluten (**A**) and gluten index (**B**) during dough proofing. Stage I, stage II, and stage III represented 20, 40, and 60 min of the first proofing period, respectively. Stage IV, stage V, stage VI, and stage VII represented 20, 40, 60, and 80 min of the second proofing period, respectively. Different capital letters on the same line indicated significant difference (*p* < 0.05) between different proofing stage at the same dosage of hemicellulase, and different lowercase letters indicated significant different (*p* < 0.05) between a different dosage of hemicellulase at the same proofing stage.

As shown in Figure 3A, the addition of enzymes led to an increase in content of wet gluten than that of the control at stage I. When hemicellulase was used at a dosage of 40 mg kg^−1^ of flour, the content of wet gluten reached 42.70% as compared to that of the control. However, the content of wet gluten of dough prepared with hemicellulase showed the opposite trend as compared to that of the control in first proofing period (stage I to stage III). The wet gluten content of dough containing hemicellulase was lower than that of the control at stage III. Gómez et al. [3] explained the negative effects of fibrous materials that may pierce gas cell walls. Physical hindrance of gluten network by the introduction of particles on gluten network matrix was also postulated. In the meantime, the contact between fiber matrix and gluten increased with the punching operation of the dough in the first proofing period. This might have weakened the gluten network, adversely affecting the content of wet gluten. In the second proofing period, application of hemicellulase at each dosage revealed a higher growth rate of wet gluten content than that of the control, but a dosage at 60 mg kg^−1^ of flour provided lower content of wet gluten than that of the control in each stage. As shown in Figure 3B, the addition of enzyme led to the rise in gluten index as compared with that of the control at stage I. Following the stage of proofing, application of hemicellulase at dosage of 20 and 40 mg kg^−1^ of flour revealed a similar trend to that of the control. Meanwhile, the gluten index was higher than that of the control at stage VII. In contrast, gluten index with a high hemicellulase dosage (60 mg kg^−1^ of flour) showed a downward trend and a lower gluten index than that of the control, which was also observed at stage VII. This result showed that the slight deterioration of dough rheological properties was not caused by the decrease in gluten quality. However, the addition of overdosage enzyme (60 mg kg^−1^ of flour) had a significant weakening effect on the content of wet gluten and gluten index, so the rheological properties of the dough was significantly reduced, which consequently led to the collapse of bread.

### 3.5. Effect of Hemicellulase on the Content of Free Sulfhydryl and Disulfide Bond during Dough Proofing

**Figure 4:**
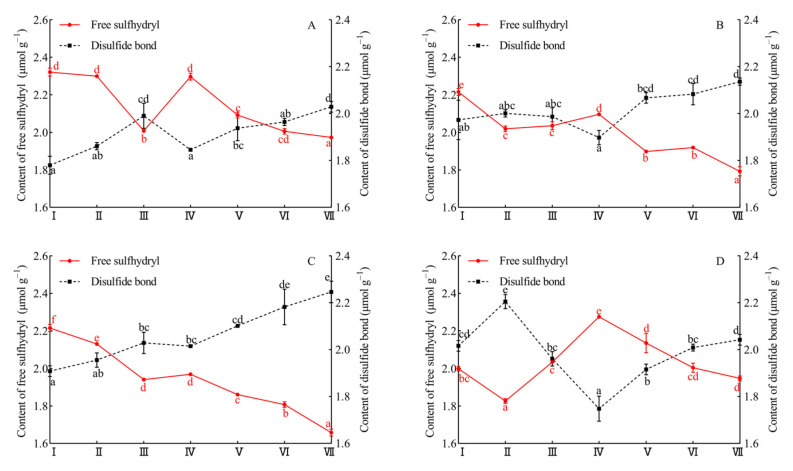
The effect of hemicellulase on the content of free sulfhydryl and disulfide bond in dough proofing. Control (**A**), 20 mg kg^−1^ of flour hemicellulase (**B**), 40 mg kg^−1^ of flour hemicellulase (**C**), 60 mg kg^−1^ of flour hemicellulase (**D**). Stage I, stage II, and stage III represented 20, 40, and 60 min of the first proofing period, respectively. Stage IV, stage V, stage VI, and stage VII represented 20, 40, 60, and 80 min of the second proofing period, respectively Different lowercase letters on the same line indicated significant differences between the different proofing stage (*p* < 0.05).

The reaction of disulfide bond and free sulfhydryl exchange is important for the formation of gluten network, so the content of disulfide bond and free sulfhydryl in dough play an important role in the processing performance of flour [44]. As shown in Figure 4, the dosages of hemicellulase had no effect on total sulfhydryl content at different proofing stages, and total sulfhydryl content of tested sample was all around 6.03 μmol g^−1^. At enzyme dosages of 20 and 40 mg kg^−1^ of flour, the disulfide bond content of the dough at stage I and the rising rate of disulfide bond content from the stage I to stage VII were higher than that of the control. Tatsuya et al. [46] mentioned that the hydrolytic activity of hemicellulase eliminated the spatial barrier of gluten protein contact and promoted the formation of disulfide bonds. In contrast, the addition with 60 mg kg^−1^ of flour revealed higher disulfide bond content then that of the control at stage I, but it provided similar disulfide bond content to that of the control at stage VII. Those results showed that excessive enzymatic hydrolysis promoted the formation of disulfide bonds at early proofing, but low molecular weight WEAX gradually accumulated with the elongation of proofing time; these molecules could combine with free sulfhydryl in gluten to form a WEAX-gluten mixed system, which may interfere with the formation of disulfide bonds in gluten [43]. Therefore, optimization of enzyme usage level was important, and fermentation conditions (fermentation time, fermentation temperature, and fermentation humidity) also need to be adjusted to prevent loaf instability due to over proofing.

### 3.6. Effect of Hemicellulase on the Content of Non-Covalent Bonds during Dough Proofing

**Figure 5:**
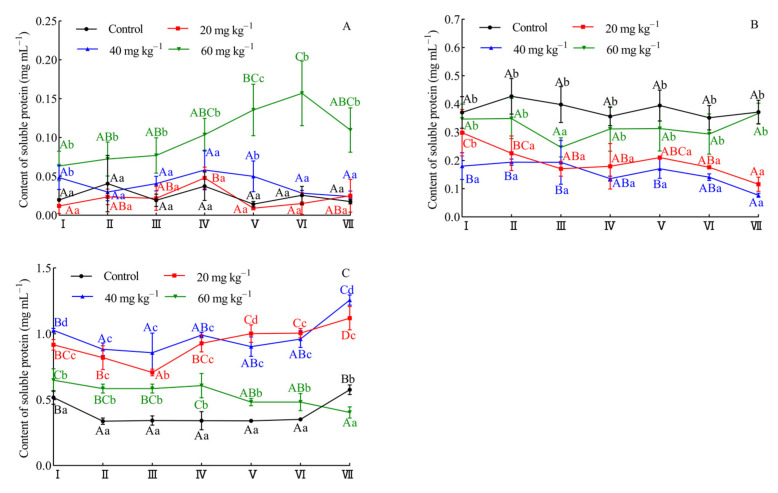
The effect of enzyme addition and proofing stage on the non-covalent bonds of dough. The contribution of ionic bonds (**A**), hydrogen bonds (**B**), and hydrophobic interactions (**C**). Stage I, stage II, and stage III represented 20, 40, and 60 min of the first proofing period, respectively. Stage IV, stage V, stage VI, and stage VII represented 20, 40, 60, and 80 min of the second proofing period, respectively. Different capital letters on the same line indicated significant difference (*p* < 0.05) between different proofing stage at the same dosage of hemicellulase, and different lowercase letters indicated significant differences (*p* < 0.05) between a different dosage of hemicellulase at the same proofing stage.

As shown in Figure 5A, dough samples supplemented with 20 and 40 mg kg^−1^ of flour had very similar contribution of ionic bonds to that of the control at each proofing stage. However, the contribution of ionic bonds at a dosage of 60 mg kg^−1^ of flour was sharply raised. This result may be due to the degree of hydrolysis of the aleurone layer cell wall which did not show an absolute linear relationship with the amount of hemicellulase used. The addition of a high dosage (60 mg kg^−1^) of hemicellulase could sharply increase the content of soluble pentosans (Figure S1); this indicated that the aleurone layer cell wall was significantly ruptured and that a large amount of content flowed out in this state. Consequently, the contribution of ionic bonds was significantly different from those of other enzyme dosages (20 and 40 mg kg^−1^). Figure 5B showed that addition of hemicellulase significantly reduced the contribution of hydrogen bonds (*p* < 0.05), but the hydrogen bond changed slightly in different stages. According to Nawrocka et al. [47], the presence of the dietary fiber during preparation of the bread dough interfered with the formation of proper gluten network by catalyzing new hydrogen bonds and caused changes in the conformation of disulphide bridges as well as in the micro-environment of two aromatic amino acids (tryptophan and tyrosine). Therefore, the hydrolysis of NSPs by hemicellulose may result in the reduction in hydrogen bonds in the dough system. However, the WEAX could also crosslink to free sulfhydryl of gluten in a WEAX-gluten mixed system, through reacting with the double bond of FA of WEAX by electrophilic addition or free-radical addition [44]. Thus, the contribution of hydrogen bonds was intensified at a dosage of 60 mg kg^−1^ of flour, which might be due to enrichment of WEAX caused by hemicellulase. As illustrated in Figure 5C, the addition of enzyme caused an increase in the contribution of hydrophobic interactions, as compared to that of the control in stage I. Following the stage of proofing, the hydrophobic interactions showed a gradual upward trend at dosage of 20 and 40 mg kg^−1^. When the added amount of hemicellulase increased further, hydrophobic interaction indicated a downward trend and a lower hydrophobic interaction than that of the control was also obtained at stage VII. It was worth noting that the influence of enzyme dosage and proofing stage on the hydrophobic interactions of the dough was very similar to the gluten index change regularity (Figure 3B), which also suggested that the gluten quality was closely related to the change of hydrophobic interactions in the dough.

### 3.7. SDS-PAGE Profiles of Gluten

**Figure 6:**
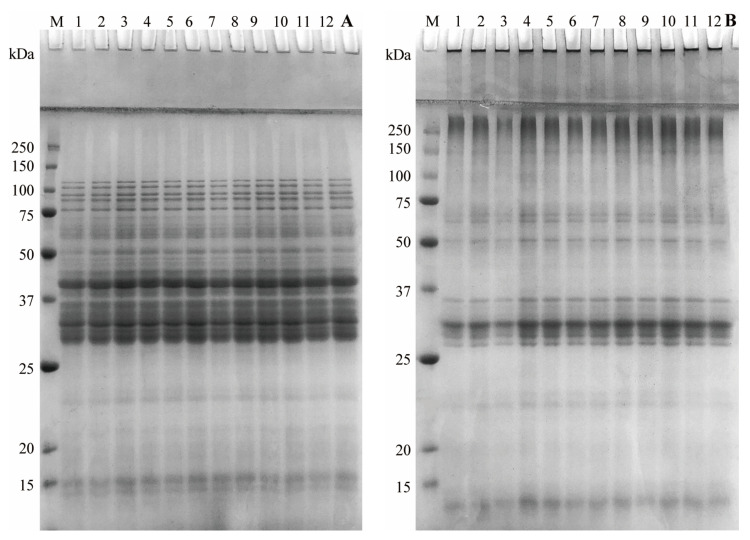
Reducing (**A**) and non-reducing (**B**) SDS-PAGE pattern of gluten in the different dough proofing stage treated by a different enzyme dosage. Lane M: pre-stained marker; lanes 1–4: gluten prepared from dough addition with 0, 20, 40, and 60 mg kg^−1^ flour enzyme dosage at stage I (20 min of proofing time in the first proofing period); lanes 5–8: gluten prepared from dough addition with 0, 20, 40, and 60 mg kg^−1^ flour enzyme dosage at stage IV (20 min of proofing time in the second proofing period); lanes 9–12: gluten prepared from dough addition with 0, 20, 40, and 60 mg kg^−1^ flour enzyme dosage at stage VII (80 min of proofing time in the second proofing period).

The influences of the proofing stage and hemicellulase dosage on gluten were tested in reducing SDS-PAGE and non-reducing SDS-PAGE conditions, as shown in Figure 6. It was clearly observed that the dosage of enzyme and proofing stage had no visible difference on SDS-PAGE profiles of gluten. Liu et al. [17] observed that protein fractions of dough liquor treated by xylanase had no significant change, although xylanase (60 mg kg^−1^ of flour) provided remarkable enhancement in content of free sulfhydryl. In a study performed by Liu et al. [48], the addition of pentosanase did not cause any change in protein fractions, but it provided higher free sulfhydryl content than that of the control. These results indicated that hydrolysis of NSPs did not affect subunits in the molecular weight of gluten. Therefore, the improvement impact of hemicellulase on wheat aleurone-rich bread still needed to be further evaluated in combination with the secondary structure of gluten.

### 3.8. Molecular Weight and Distribution of Gliadins

**Figure 7:**
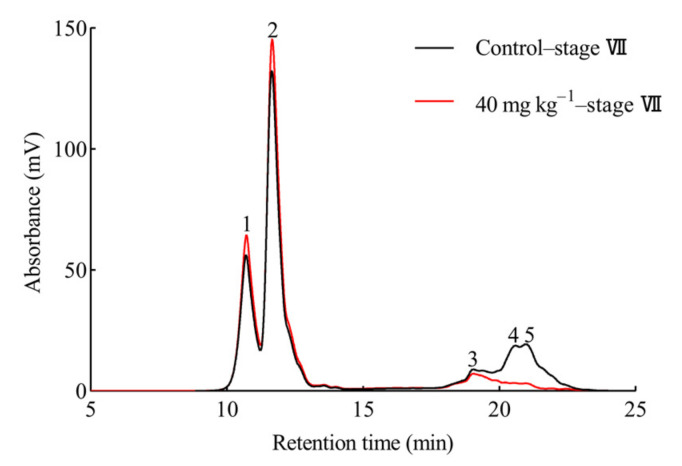
The effect of hemicellulase on molecular weight and distribution of gliadins at stage VII. Stage VII (80 min of proofing time in the second proofing period). According to the retention time from short to high, the chromatographic peaks were named peak 1 to peak 5, respectively.

The effect of hemicellulase on molecular weight and distribution of gliadins at stage VII was determined by GPC. It can be seen from Figure 7 that there were five peaks for gliadin of the control group and the hemicellulase-treated group; the retention time of each elution peak in the control group was 10.71, 11.65, 19.04, 20.59, and 20.98 min, respectively; and the retention time of each elution peaks in the hemicellulase-treated group were 10.72, 11.66, 19.05, 19.87, and 20.99 min, respectively. Similar retention times indicate that hemicellulase did not affect the molecular weight of gliadin components. The relative area of each elution peak (peak 1 to peak 5) in the control group was 21.87%, 52.93%, 5.58%, 8.63%, and 10.99%, respectively, and the relative area of each elution peak (peak 1 to peak 5) in the hemicellulase-treated group was 27.32%, 64.68%, 4.64%, 2.11%, and 1.26%, respectively. Those results suggested that the hydrolytic activity of hemicellulase led to a considerable decrease in the number of lower molecular weight oligomers which presumably arose from polymerization of gliadins. By combining the characteristics of bread and dough in the present study, the aggregation of gliadin could improve the extensibility of the dough and increase the specific volume of the bread.

### 3.9. Secondary Structure of Gluten

**Figure 8:**
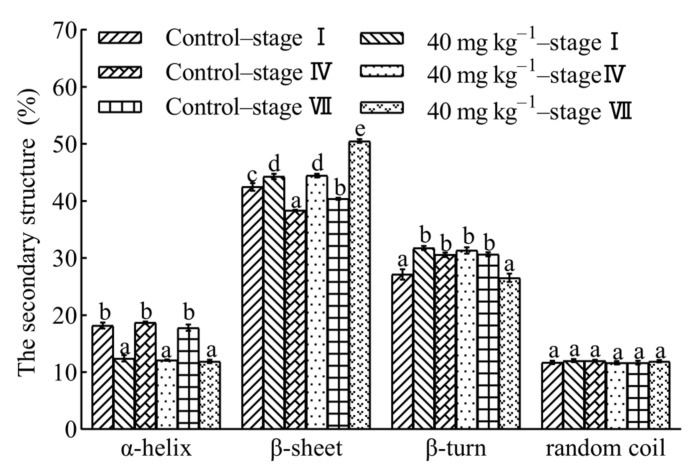
The effect of hemicellulase on secondary structure of gluten during proofing. Stage I (20 min of proofing time in the first proofing period), stage IV (20 min of proofing time in second proofing period), and stage VII (80 min of proofing time in the second proofing period). Different lowercase letters indicated significant different in the same structure (*p* < 0.05).

The effect of enzyme on the secondary structure of gluten in different proofing stages at a dosage of 40 mg kg^−1^ of flour is shown in Figure 8. The addition of hemicellulase significantly reduced the proportion of α-helix (*p* < 0.05), but the proofing stages had no effect on the content. This result showed that hemicellulase limited the aggregation of gluten polymer and facilitated gluten network more stretchable [46]. These results explained that the extension of dough supplemented with hemicellulose (40 mg kg^−1^) was higher than that of the control. The proportion of β-sheet significantly enhanced at a dosage of 40 mg kg^−1^ of flour, and the growing rate of proportion of β-sheet gradually raised with proofing. It had been stated by Alpers et al. [49] that an increase in the proportion of β-sheet indicate good gluten viscoelasticity and baking quality. Moreover, the addition of hemicellulase significantly reduced the α-helix/β-sheet ratio in each proofing stage (Figure S2). At the same time, the addition of hemicellulase (40 mg kg^−1^) promoted the formation of disulfide bonds in the dough and enhanced the contribution of hydrophobic interactions as compared to that of the control; however, the subunits molecular weight of gluten did not change. This evidence confirmed that the hydrolysis activity of hemicellulase affected the secondary structure of gluten protein by changing the intermolecular forces but not through the molecular weight of protein subunits. [46]. The proportion of β-turn of gluten prepared with hemicellulase was lower than that of the control at stage VII, although the addition of hemicellulase caused an increase in the proportion of β-turn. In contrast, proofing stages (stage I, stage IV, and stage VII) and dosages of hemicellulase (0 and 40 mg kg^−1^ of flour) had no influence on the proportion of random coil. Thus, the result of the present study indicated that a high proportion of β-sheet, together with low proportion of α-helix and β-turn, may occur a satisfactory relationship with the improvement of wheat aleurone-rich bread quality [45,50].

## 4. Conclusions

In this research, the effect of hemicellulase addition on the bread quality and rheological properties of wheat aleurone-rich flour were determined. The results showed that incorporation hemicellulase into wheat aleurone-rich bread formulations led to considerably higher specific volume and lower crumb firmness than those of the control. The enhancement of dough handling and bread quality was due to hemicellulase which could improve the gluten quality during the proofing of dough, although the rheological performances of the wheat aleurone-rich dough were slightly reduced at this time. In other words, the deterioration of the rheological properties may be due to decreasing consistency of dough system caused by the hydrolysis of NSPs. The intermolecular forces indicated that the improvement of the gluten network was due to the enhanced disulfide bonds and hydrophobic interactions, and the decrease in hydrogen bonds, while the ionic bond had a slight influence on it. At an enzyme dosage of 40 mg kg^−1^ of flour, the proportion of α-helix of each proofing stage (stage I, stage VI, and stage VII) was significantly lower than that of the control, and the proportion of β-sheet was remarkably higher than that of the control. These results suggest that the viscoelasticity of gluten was enhanced. On the other hand, we also observed that the dough supplemented with 60 mg kg^−1^ of flour had the largest proof volume, but collapsed during baking. According to the existing research, it could be determined that this phenomenon is directly related to the decline in gluten quality. However, further exploration is required with regards to which kind of specific hemicellulase hydrolysates accumulates will destroy the gluten network.

## Figures and Tables

**Table 1:** Effect of different dosages of hemicellulase preparations on farinograph properties of dough.

Dosage of Enzyme Added (mg kg^−1^)	Farinograph Parameters			
	WA (%)	DDT (min)	ST (min)	DS (UF)
Control	67.47 ± 0.25 a	6.26 ± 0.35 b	12.70 ± 0.8 c	35.33 ± 2.08 a
20 mg kg^−1^	67.73 ± 0.21 a	6.17 ± 0.11 b	11.76 ± 0.79 bc	37.00 ± 2.65 ab
40 mg kg^−1^	67.57 ± 0.15 a	6.15 ± 0.24 b	11.23 ± 0.53 ab	40.00 ± 1.73 b
60 mg kg^−1^	67.27 ± 0.32 a	5.58 ± 0.13 a	9.88 ± 0.07 a	45.00 ± 2.65 c

A, water absorption; DDT, development time; ST, stability time; DS, degree of softening. Means with different letters in the same column correspond to a significant difference between the different dosage of hemicellulase (*p* < 0.05).

## Supplementary Materials

The following are available online at https://www.mdpi.com/article/10.3390/foods10102427/s1, Table S1: Effect of hemicellulase addition on pasting properties of wheat aleurone-rich flour, Figure S1: The effect of hemicellulase on the content of water-soluble pentosan during dough proofing, Figure S2: The effect of hemicellulase on α-helix/β-sheet ratio of gluten during proofing.
