# Supplementary material for: Monitoring the Effects of Hemicellulase on the Different Proofing Stages of Wheat Aleurone-Rich Bread Dough and Bread Quality
Source: Foods. 2021 Oct 13;10(10):2427. doi: 10.3390/foods10102427 (PMC8535788; doi:10.3390/foods10102427)
Supplement: Supplementary file 1 [file foods-10-02427-s001.zip › Figure S1The effect of hemicellulase on the content of water-soluble pentosan during dough proofing..pdf]

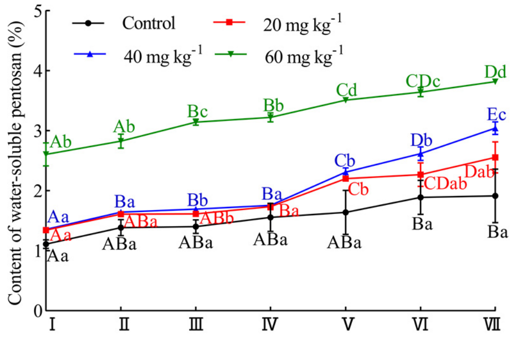

Figure S1. The effect of hemicellulase on the content of water-soluble pentosan during dough proofing. Stage I, stage II, and stage III represented 20, 40, and 60 min of the first proofing period, respectively. Stage IV, stage V, stage VI, and stage VII represented 20, 40, 60, and 80 min of the second proofing period, respectively. Different capital letters on the same line indicated significant difference ($p < 0.05$) between different proofing stage at the same dosage of hemicellulase, and different lowercase letters indicated significant different ($p < 0.05$) between different dosage of hemicellulase at the same proofing stage.
