# Supplementary material for: Monitoring the Effects of Hemicellulase on the Different Proofing Stages of Wheat Aleurone-Rich Bread Dough and Bread Quality
Source: Foods. 2021 Oct 13;10(10):2427. doi: 10.3390/foods10102427 (PMC8535788; doi:10.3390/foods10102427)
Supplement: Supplementary file 1 [file foods-10-02427-s001.zip › Figure S2 The effect of hemicellulase on a┴-helia┬-sheet ratio of gluten during proofing.pdf]

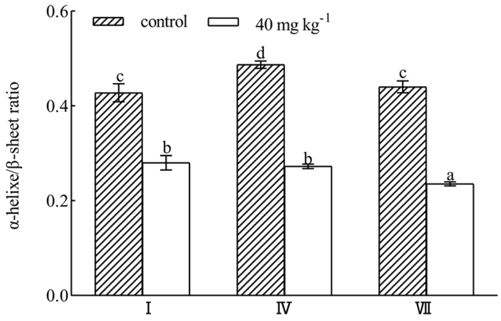

Figure S2. The effect of hemicellulase on α-helix/β-sheet ratio of gluten during proofing. Stage I (20 min of proofing time in the first proofing period), stage IV (20 min of proofing time in second proofing period), and stage VII (80 min of proofing time in the second proofing period). Different lowercase letters indicated significant different in the same structure ($p < 0.05$).
