# Supplementary material for: Monitoring the Effects of Hemicellulase on the Different Proofing Stages of Wheat Aleurone-Rich Bread Dough and Bread Quality
Source: Foods. 2021 Oct 13;10(10):2427. doi: 10.3390/foods10102427 (PMC8535788; doi:10.3390/foods10102427)
Supplement: Supplementary file 1 [file foods-10-02427-s001.zip › Table S1 Effect of hemicellulase addition on pasting properties of wheat aleurone-rich flour.pdf]

Table S1. Effect of hemicellulase addition on pasting properties of wheat aleurone-rich flour

Treatment	Pasting temperature(°C)	Peak viscosity (BU)	Final viscosity (BU)	Breakdown viscosity (BU)	Setback viscosity (BU)
Control	60.63±0.15a	1443.00±11.31b	2002.50±10.61c	397.00±2.83c	917.50±0.71c
20 mg kg^{-1}	60.87±0.31a	1367.33±15.53a	1934.00±1.41b	373.33±7.09b	888.67±6.81b
40 mg kg^{-1}	60.83±0.06a	1357.00±3.61a	1905.33±6.51ab	367.67±4.93b	872.33±4.93a
60 mg kg^{-1}	60.90±0.10a	1333.00±14.14a	1880.50±7.78a	346.50±3.54a	863.50±7.78a

Means with different letters in the same column correspond to a significant difference between the different dosage of hemicellulase ($p < 0.05$).
